# Impact of older age at Fontan completion on mid-term survival

**DOI:** 10.1186/s43044-022-00314-5

**Published:** 2022-10-15

**Authors:** Ruth Grace Aurora, Radityo Prakoso, Dicky Fakhri, Indriwanto Sakidjan, Sisca Natalia Siagian, Prima Almazini, Oktavia Lilyasari

**Affiliations:** 1 Department of Cardiology and Vascular Medicine, Faculty of Medicine, National Cardiovascular Center Harapan Kita, Universitas Indonesia, Jl. Letjen S. Parman Kav 87, Slipi, Jakarta, 11420 Indonesia; 2 Pediatric and Congenital Heart Surgery Unit, Department of Surgery, National Cardiovascular Center Harapan Kita, Jalan Let. Jend. S. Parman Kav 87, Jakarta Barat, 11420 Indonesia

**Keywords:** Age at operation, Fontan completion, Mid-term survival, Older age

## Abstract

**Background:**

The optimum age of Fontan completion remains unknown. Currently, the majority of centers worldwide are performing Fontan completion at 2–4 years of age. In Indonesia, lack of awareness and limited resources probably explain why patients seek treatment at advanced stage. This study aimed to evaluate the impact of older age at Fontan completion on mid-term survival.

**Results:**

A single-center retrospective cohort study was performed on 261 patients who underwent Fontan completion between 2008 and 2019 and survived to discharge. The patients were followed up until April 2020, with a median follow-up period of 3 years (range 0–12 years). The median age was 5 years (range 2–24 years). The survival rates of patients with the age at operation ≤ 6 years and > 6 years were 92.1% and 82.8%, respectively. A subgroup analysis showed that the survival rates for age < 4 years, 4–6 years (reference age), 6–8 years, 8–10 years, 10–18 years, and > 18 years were 85.7%, 94.8%, 85.4%, 78.8%, 85.7%, and 66.7%, respectively. Age at Fontan completion of > 6 years (HR 3.84; *p* = 0.020) was associated with a lower 12-year survival rate. The age at operation of 8–10 years (HR 6.79; *p* = 0.022) and > 18 years (HR 15.30; *p* = 0.006) had the worst survival rates.

**Conclusions:**

An older age at Fontan completion (> 6 years) significantly reduced mid-term survival rate. The age at Fontan of 8–10 years and > 18 years had higher risk of mid-term death than age of 4–6 years.

## Background

Fontan completion is a palliative surgery performed on congenital heart disease (CHD) patients with single ventricular anatomy or physiology [1, 2]. It was first performed at National Cardiovascular Center Harapan Kita (NCCHK) in 1992. In a “Fontan circulation,” the systemic veins are directly connected to the pulmonary arteries, resulting in a passive pulmonary circulation without the support of a sub-pulmonary ventricle [3, 4].

Within the last five decades, advancements in surgical and medical management have greatly improved patient outcomes after Fontan completion. Late consequences of the Fontan circulation include central venous hypertension, decreased cardiac output, hepatic dysfunction, and protein loosing enteropathy (PLE) [5, 6]. Friedrich-Rust et al. stated that these phenomena were associated with an older age at Fontan completion and the duration of Fontan circulation itself [6].

The optimal timing of Fontan completion remains controversial, and the 2019 American Heart Association (AHA) scientific statement does not provide a standardized age for Fontan completion. According to the “Ten Commandments” of Fontan by Choussat et al. the age at operation should be above 4 years old [7]. On the other hand, some studies showed that age of 2–4 years was the best timing to perform the Fontan procedure. Shiraishi et al. [1] reported than Fontan procedure performed at a young age (< 3 years) is beneficial for the mid-term exercise capacity compared to surgery performed at age ≥ 3 years [8]. Previously, a meta-analysis showed that an older age at Fontan completion was associated with higher mortality after 5, 10, and 15 years after surgery [9]. Some experts argue that postponing Fontan completion could delay the onset of Fontan complications. Fauziah et al. reported that most patients who underwent Fontan completion in Indonesia were older than patients in developed countries, with a median age of 6 years (range 3–22 years) [10].

Currently, the optimum age of Fontan completion that results in the best mid-term outcomes remains unknown. In Indonesia, lack of awareness and limited resources probably explain why patients seek treatment at a delayed stage. However, there are limited data concerning the effect of older age at Fontan completion toward mid-term survival in Indonesia.

## Methods

This was a single-center retrospective cohort study, comparing the 12-year survival after Fontan completion at < 6 years old and ≥ 6 years old. The study was approved by the institutional review board of the National Cardiovascular Center Harapan Kita Hospital. Records of all patients who underwent Fontan procedure in our hospital from January 2008 to December 2019 were reviewed. Baseline demographic, anatomic, preoperative, and perioperative data were extracted from the registry, pediatric surgical conferences, medical records, surgery reports, echocardiography and catheterization reports, and follow-up data were collected until the end of the study period (April 2020). Time-to-event analysis began from the first day of hospital discharge after the Fontan completion. Patients who died during surgery or postoperative hospitalization were excluded.

A total of 284 patients with functionally single-ventricle anatomy underwent Fontan completion during the study period. Patients who met the inclusion criteria were divided into two different groups; age at Fontan completion (operation) < 6 and ≥ 6 years old. This age cutoff was based on a previous average age for Indonesian Fontan patients by Fauziah et al. [10]. Clinical follow-up was completed by medical record review, phone calls, or mails.

The data were evaluated for death as the primary end-point. Preoperative factors, intraoperative factors, perioperative morbidities, length of stay, rehospitalization, and medical therapy were analyzed as secondary endpoints. The medication was the last drugs prescribed at the end of the study. The patients were classified as having the event of death or being censored at the end of the study period. The median follow-up period was 3 years (range 0–12 years). Fontan completion was defined as any form of total cavopulmonary connection including a lateral tunnel or an extracardiac conduit. No atriopulmonary connections were performed at our center during this period.

All statistical analyses were performed using IBM SPSS version 24.0 software (SPSS, Inc., Chicago, IL, USA). Categorical variables are summarized with frequencies and percentages. Skewed variables are summarized as median and range or interquartile range. The survival analysis was performed using the Kaplan Meier curve analysis. The differences between age at operation groups were compared with Kaplan Meier Log Rank (Mantel Cox) with a *p* < 0.05 considered statistically significant. Bivariate analysis of risk factors for overall mortality with a *p* < 0.25 in Log Rank Test will be continued with a backward stepwise multivariate cox regression analysis to determine the final model that can predict mid-term survival.

## Results

**Fig. 1:**
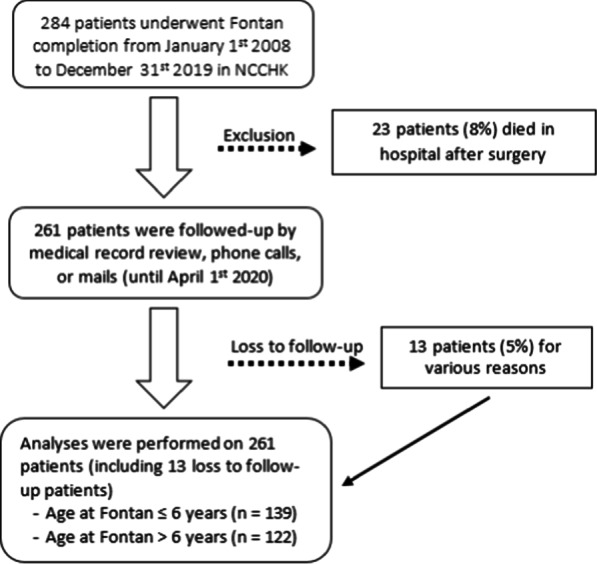
Study flow chart

Tables 1 and 2 show baseline characteristic of this study profile at the completion of study. 261 patients discharged from the hospital after the Fontan surgery were enrolled; 139 patients (53.5%) were ≤ 6 years of age and 122 patients (46.7%) were > 6 years of age. Thirteen patients (5%) dropped out from the study for various reasons. The study flow chart can be seen in Fig. 1.

**Table 1:** Baseline characteristics of 261 patients underwent a Fontan completion

Variable	Age ≤ 6 years (*n* = 139)	Age > 6 years (*n* = 122)	*p* value
Sex			0.964
Female (*n* = 113)	60 (43.2%)	53 (43.4%)	
Male (*n* = 148)	79 (56.8%)	69 (56.6%)	
Systemic ventricle morphology			0.084
LV morphology (*n* = 122)	58 (41.7%)	64 (52.5%)	
RV morphology (*n* = 139)	81 (58.3%)	58 (41.7%)	
mPAP (mmHg) [range]	10 [4–16]	11 [4–18]	0.275
LVEDP (mmHg) [range]	10 [4–17]	10 [3–16]	0.448
Preoperative saturation (%) [range]	81 [53–98]	79 [60–98]	0.029*
≤ 80% (*n* = 135)	64 (46%)	71 (58.2%)	0.050
> 80% (*n* = 126)	75 (54%)	51 (41.8%)	
Type of pulmonary blood flow			0.117
Restricted PBF (*n* = 189)	95 (68.3%)	94 (77%)	
Unrestricted PBF (*n* = 72)	44 (31.7%)	28 (23.0%)	
AV valve regurgitation			0.486
Yes (*n* = 34)	20 (14.4%)	14 (11.5%)	
No (*n* = 227)	119 (85.6%)	108 (88.5%)	
Prior BCPS procedure			0.253
Primary Fontan (*n* = 34)	15 (10.8%)	19 (15.6%)	
Staging Fontan (BCPS) (*n* = 227)	124 (89.2%)	103 (84.4%)	
Duration between BCPS and Fontan (years)	2 (1–5)	5 (0–17)	< 0.001*
≤ 3 years (*n* = 166)	92 (66.2%)	74 (60.7%)	< 0.001*
> 3 years (*n* = 95)	47 (33.8%)	48 (39.3%)	
*Intraoperative factors*			
Type of Fontan connection			0.396
LT (*n* = 14)	9 (6.5%)	5 (4.1%)	
ECC (*n* = 247)	130 (93.5%)	117 (95.9%)	
Fenestration			0.086
Yes (*n* = 181)	90 (64.7%)	91 (74.6%)	
No (*n* = 80)	49 (35.3%)	31 (25.4%)	
Duration of AoX (minutes) [range]	0 (0–198)	25 (0–131)	0.001*
Duration of CPB (minutes) [range]	107 (36–340)	136 (28–543)	< 0.001*
CPB ≤ 120 min (*n* = 128)	85 (61%)	43 (35%)	< 0.001*
CPB > 120 min (*n* = 133)	54 (39%)	79 (65%)	
*Postoperative factors*			
Length of stay (days) [range]	15 (6–86)	14,5 (6–97)	0.588
LOS ≤ 15 days	70 (50.4%)	67 (54.9%)	0.463
LOS > 15 days	69 (49.6%)	55 (45.1%)	
Duration of Fontan circulation (years)			0.355
0–3 years	92 (66.2%)	74 (60.7%)	
> 3 years	47 (33.8%)	48 (39.3%)	

*LV* left ventricle, *RV* right ventricle, *mPAP* mean pulmonary arterial pressure, *LVEDP* left ventricular end-diastolic pressure, *PBF* pulmonary blood flow*, AV* atrioventricular, *BCPS* bidirectional cavopulmonary shunt, *LT* lateral tunnel, *ECC* extracardiac conduit, *AoX* aortic cross-clamping time, *CPB* cardiopulmonary bypass, *LOS* length of stay

*p < 0.05: significantly different between two groups

**Table 2:** Baseline postoperative morbidity and medication characteristics of 261 patients underwent a Fontan completion

Postoperative morbidity	Age ≤ 6 years (*n* = 139)	Age > 6 years (*n* = 122)	*p* value
Thromboembolism	8 (5.8%)	9 (7.4%)	0.597
PLE	0 (0%)	8 (6.6%)	0.002*
Pleura effusion	68 (48.9%)	62 (50.8%)	0.760
Postoperative arrhythmia	40 (28.6%)	30 (24.6%)	0.447
Infection	35 (25.2%)	27 (22.1%)	0.815
Heart failure	3 (2.2%)	9 (7.4%)	0.045*
Bleeding	18 (12.9%)	13 (10.7%)	0.568
Rehospitalization	38 (27.3%)	33 (27%)	0.958
Medication**			
ACE inhibitor	113 (81.3%)	90 (73.8%)	0.145
Sildenafil	56 (40.3%)	45 (36.9%)	0.574
Furosemide	26 (18.7%)	16 (13.1%)	0.221
Antiplatelet	9 (6.5%)	10 (8.2%)	0.594
Anticoagulant	126 (90.6%)	102 (83.6%)	0.088
Beta blocker	19 (13.7%)	15 (12.3%)	0.743

*PLE* protein losing enteropathy, *ACE* angiotensin-converting enzyme

*p < 0.05: significantly different between two groups

**Medication which still prescribed until the end of study period

Regarding Table 1, the preoperative saturation in both groups was significantly different (*p* = 0.029), where the median preoperative saturation was significantly lower in a group age > 6 years (79%; range 60–98%). Our study found that the duration between BCPS and Fontan in both groups was significantly different (*p* < 0.001), where the group age at Fontan ≤ 6 years had a mean duration of BCPS—Fontan of 2 years (range 1–5 years) and a group of age > 6 years had a mean duration of 5 years (range 0–17 years). The mean duration between BCPS and Fontan ≤ 3 years was found significantly more in a group age ≤ 6 years. The median duration of CPB in both groups was also significantly different (*p* < 0.001), the group of age ≤ 6 years had a shorter duration of CPB time 107 (range 36–340) minutes, while the group of age > 6 years had a longer of median duration of CPB time 136 (range 28–543) minutes.

**Fig. 2:**
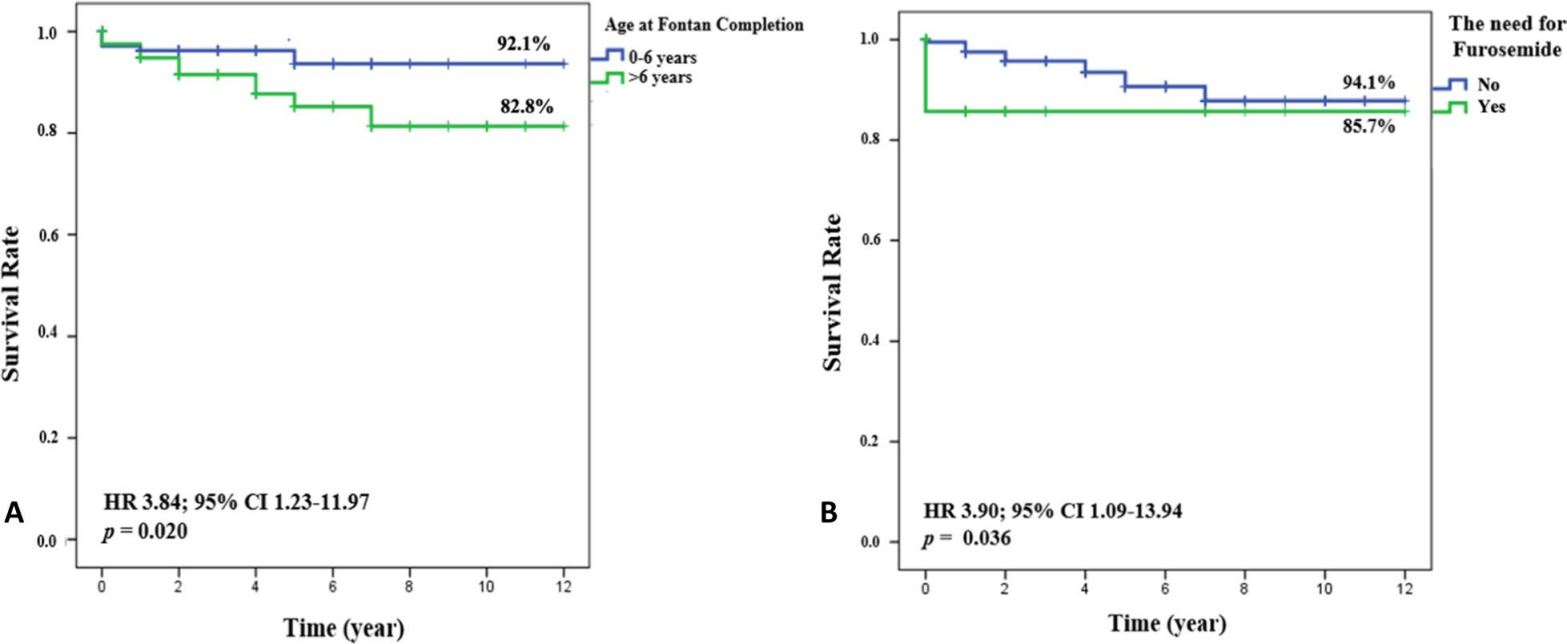
**A** Kaplan–Meier survival curve based on age at Fontan completion and **B** based on the need for furosemide until the end of study period. HR: hazard ratio; CI: confidence interval. *p* < 0.05: statistically significant

The median follow-up period was 3 years (range 0–12 years). The overall survival rate was 87.7%. Multivariate analysis was performed on variates with a *p* < 0.25 on bivariate analysis, including staging or primary Fontan, the CPB duration, length of stay (LOS), thromboembolic events, PLE, pleural effusion, arrhythmia, infection, heart failure, rehospitalization, use of an ACE-I, sildenafil, furosemide, antiplatelet, anticoagulant, beta blocker, as well as age at Fontan completion. There were significant differences in 12-year survival between age at Fontan completion ≤ 6 years and > 6 years and also the need for furosemide after Fontan completion, as shown in Table 3 and Fig. 2. The need for furosemide means use of furosemide documented until the end of study period.

**Table 3:** Multivariate analysis of factors affecting 12-year survival after Fontan completion

Variable	HR	95% CI	*p* value
Age at Fontan > 6 years	3.84	1.23–11.97	0.020*
The need for furosemide	3.90	1.09–13.94	0.036*
Postoperative heart failure	3.83	0.90–16.24	0.068
*Primary* Fontan	2.40	0.64–8.92	0.191

*HR* hazard ratio; *CI* confidence interval

*p < 0.05: statistically significant

**Fig. 3:**
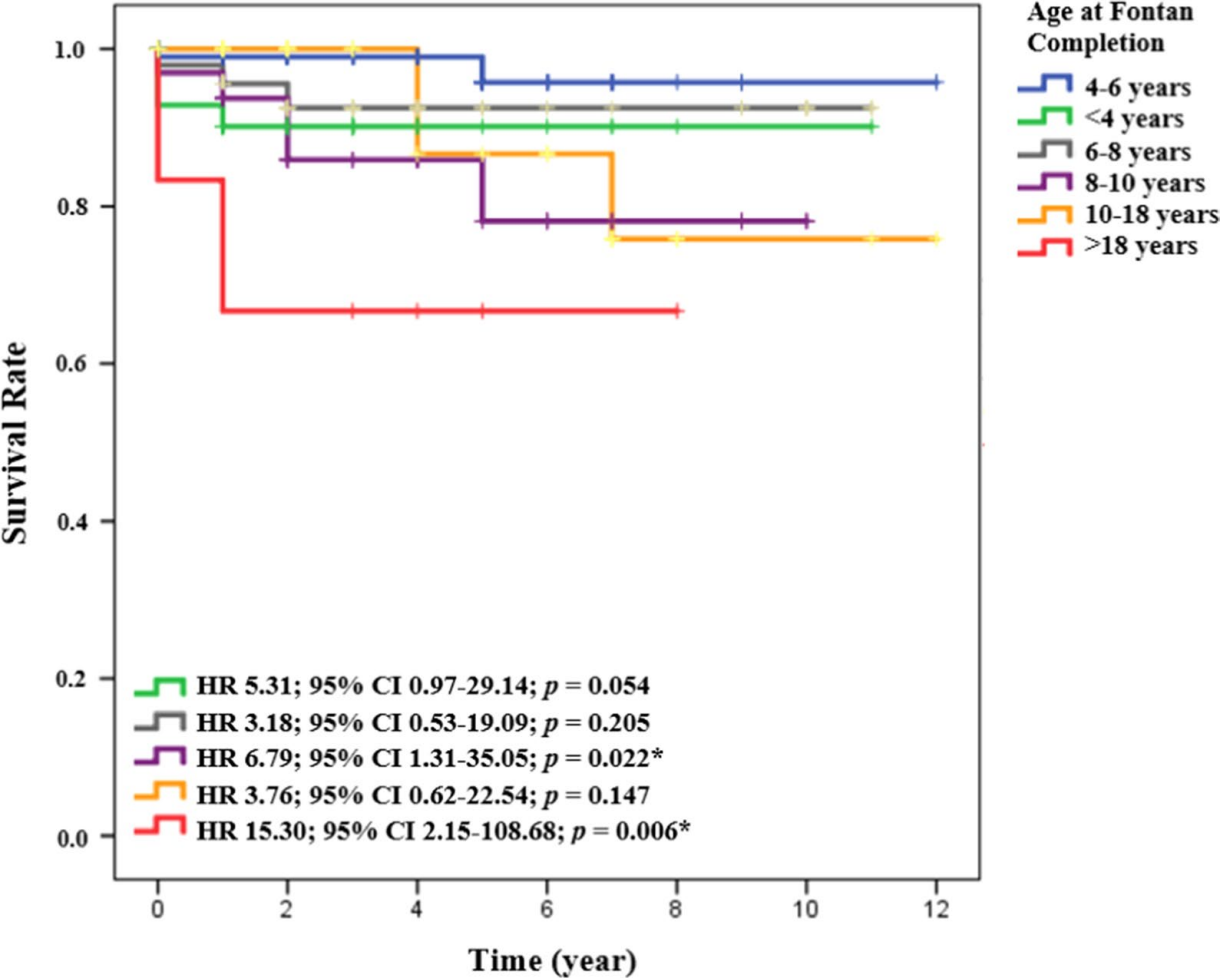
Kaplan–Meier survival curve based on subgroup analysis of age at Fontan completion. HR: hazard ratio; CI: confidence interval. *p < 0.05: statistically significant

Older age (> 6 years) at Fontan completion was associated with significant reduction in the mid-term survival, and increased the mortality risk by 3.29 times. We categorized the age at Fontan completion further into 6 subgroups: (1) < 4 years, (2) 4–6 years (reference age), (3) 6–8 years, (4) 8–10 years, (5) 10–18 years, and (6) > 18 years. The results of cox regression analysis showed that age > 18 years had the lowest survival rate (66.7%), with a 15.3 times risk of mid-term mortality compared to age of 4–6 years (Table 4). The age at Fontan completion with the lowest survival rates were (1) > 18 years (*p* = 0.006), (2) 10–18 years (*p* = 0.147), (3) 8–10 years (*p* = 0.022) (Fig. 3).

**Table 4:** Subgroup analysis of age at Fontan completion: (1) < 4 years, (2) 4–6 years, (3) 6–8 years, (4) 8–10 years, (5) 10–18 years, (6) > 18 years

Age at Fontan	*n* (%)	Survival rate	HR	95% CI	*p* value
4–6 years*	97 (37.2%)	94.8%			
< 4 years	42 (16.1%)	85.7%	5.31	0.97–29.14	0.054
6–8 years	48 (18.4%)	85.4%	3.18	0.53–19.09	0.205
8–10 years	33 (12.6%)	78.8%	6.79	1.31–35.05	0.022**
10–18 years	35 (13.4%)	85.7%	3.76	0.62–22.54	0.147
> 18 years	6 (2.3%)	66.7%	15.30	2.15–108.68	0.006**

The data were analyzed using *cox regression*. Age of 4–6 years as a reference age

*HR* hazard ratio; *CI* confidence interval

*reference age

**p < 0.05: statistically significant

## Discussion

According to this study, older age at operation significantly reduced mid-term survival after Fontan completion. The median age of Fontan completion was 5 years (range 2–24 years), similar to the previous Indonesian study by Fauziah et al. [10]. However, several studies in developed countries have reported that Fontan procedures are mostly performed at a younger age. Akintoye et al. showed that in the United States, Fontan surgery was mostly performed at an age of 2 years. [11]. A systematic review by Kvernaleand et al. reported that the Fontan procedure is conducted at a much younger age in the post-1990 era (median age 2 years, mean age 5.6 years) [12]. In Indonesia, a lack of awareness and limited resources are probably the reasons behind delayed medical treatment for CHD.

The majority of subjects in this study (87%) underwent Fontan staging (with a previous BCPS procedure), which could delay the timing of Fontan completion. This is a plausible explanation for older age at Fontan completion in the Fontan staging group. Yi et al. investigated the mortality and morbidity based on the duration between BCPS and Fontan completion > 3.7 years and < 3.7 years. [13] They found that group with longer duration between BCPS and Fontan (> 3.7 years) had an older mean age of Fontan completion (8.24 ± 3.82 years) compared to group with shorter duration of BCPS and Fontan (< 3.7 years) [13]. However, no significant difference in mortality and morbidity was found between both groups [13]. In our study, the mean BCPS-Fontan duration was significantly shorter in patients with a Fontan completion at ≤ 6 years compared to > 6 years (2 years vs 5 years, p < 0.001). This is consistent with the study by Yi et al. [13].

On multivariate analysis, age at Fontan completion > 6 years (HR 3.84; 95% CI 1.23–11.97; *p* = 0.020) and the need for mid-term furosemide (HR 3.90; 95% CI 1.09–13.94, *p* = 0.036) were significant predictors of mid-term mortality. The survival rate in patients requiring furosemide until the end of the study period was 85.7%, and in those not requiring furosemide was 94.1% (Fig. 2B). Alsaied et al. published Fontan long-term mortality risk scoring system, which considered the need for diuretics (furosemide) as a risk factor with a score of 7 (HR 1.58–9.16) [14]. It was suggested that furosemide consumption was associated with heart failure [15]. Our study found that mid-term need for furosemide after surgery was associated with higher mortality, likely due to the heart failure itself.

In the multivariate analysis, presence of heart failure increased the risk of mortality, although this was not statistically significant (HR 3.83; 95% CI 0.90–16.24; *p* = 0.68). As the assessment of heart failure in our study was only based on clinical parameters, some diagnoses might have been missed. Based on ESC and AHA guidelines, a cardiac MRI is the gold standard in evaluating the ventricular function of patients after Fontan surgery [1, 15]. Furthermore, the AHA guidelines recommended regular cardiac MRI evaluation every 2–3 years after the Fontan surgery [1].

The survival rate in the group with age at Fontan completion ≤ 6 years was 92.1%, and in the > 6 years group was 82.8% (Fig. 2A). In comparison, a survival study by d’Udekem et al. investigated the association of increased age at Fontan completion with short-term survival: age at Fontan procedure > 7 years had higher short-term mortality (HR 2.7; 95% CI 1.2–5.7; *p* = 0.012) compared to age 3–5 years [16]. Older age of Fontan completion was associated with adverse effects of prolonged ventricular overload as well as prolonged persistent cyanosis resulting in worsening myocardial function [17]. Furthermore, the relationship between early mortality and age at Fontan completion formed a U-shaped curve as reported by Akintoye et al. and Iyengar et al. [11, 17].

Thus, we performed a subgroup analysis by dividing the patients into 6 age groups (Fig. 3). It was found that subgroups of age at Fontan completion > 8 years (including 8–10 years, 10–18 years, and > 18 years) was associated with the lowest survival rate compared to other age groups. Moreover, age at Fontan > 18 years had the lowest survival, even from the first year of follow-up (Fig. 3). Age at Fontan completion > 18 years had a mid-term mortality risk of 15.3 times (*p* = 0.006) with a 12-year survival rate of 66.7%. Hence, we suggest that prior to performing Fontan completion on candidates over 18 years old, the risks, benefits, and comorbidities should be considered.

Some experts consider that performing Fontan completion in early childhood would avoid the consequences of prolonged cyanosis and systemic ventricle volume overload, as well improve postoperative functional capacity. The oxygen consumption and exercise capacity progressively decrease in all patients undergoing the Fontan operation, regardless of whether they are operated early or late. Moreover, Ovroutski et al. found that in patients who underwent the Fontan procedure at an age > 18 years, their functional capacity significantly was significantly reduced compared to patients who underwent the Fontan procedure in childhood (< 13 years) and adolescence (13–18 years) [18]. The functional capacity of patients operated upon in childhood seemed to be more stable [19].

At an early follow-up, patients with the age at Fontan completion 10–18 years had good survival rates. However, after 4 years of follow-up, the survival rate of this group declined until the end of the study (Fig. 3). It was similar to the study by Ono et al. showing that 15-year survival rate in older age group (pre-adolescents, adolescents, and adults) decreased to 86.5%, compared to age at Fontan < 9 years, which had a survival rate of 94% [19]. Ono et al. demonstrated that AV valve regurgitation was closely associated with ventricular function [19]. A competent AV valve is a prerequisite for ventricular function recovery [19]. The proportion of preoperative AV valve regurgitation in group of age 8–10 years and 10–18 years were 15.2 and 11.4%, respectively. We believe that this smaller proportion of AV valve regurgitation was probably the reason for better survival rates in the 10–18 years group compared to the 8–10 years group, despite being older.

In addition, subgroup analyses of heart failure incidence based on age at Fontan found that the proportion of heart failure was lower in 10–18 years group than the 8–10 years group (16.7% vs 25%)—another possible reason for the better survival rate in older group. Hence, we did not find an exact inverse relationship between age at Fontan completion and survival rate, probably due to the influence of other factors such as heart failure and preoperative AV valve regurgitation.

## Limitation of the study

The data collection in our retrospective study depends on the past accuracy and comprehensiveness of medical documentation. The follow-up duration for each patient varies from 0 to 12 years, and hence some events may not have occurred yet in patient with shorter follow-up duration. We did not have the opportunity to assess postoperative ventricular function using the gold standard cardiac MRI, which could be a potential source of bias.

## Conclusions

Older age (> 6 years) at Fontan completion decreased mid-term survival and increased the mortality risk by 3.29 times. The age at Fontan completion of 8–10 years and > 18 years had higher risk of mid-term mortality (6.7 times and 15.3 times) than age of 4–6 years. Therefore, consideration of the risks, benefits, and the comorbidities is necessary in those Fontan candidates who are > 6 years old.

## Data Availability

All data generated or analyzed during this study are included in this published article.

## Abbreviations

BCPS: Bidirectional cavopulmonary shunt
LT: Lateral tunnel
ECC: Extracardiac conduit
AoX: Aortic cross-clamping time
CPB: Cardiopulmonary bypass
LOS: Length of stay
PLE: Protein losing enteropathy
ACE: Angiotensin-converting enzyme
